# A Case of Injury to the Hand from a Hay-Cutting Machine

**Published:** 1858-08

**Authors:** 


ART. VI.—*A Case of Injury to the Hand from a Hay-cutting Machine, in which the Index and Middle Fingers of the Left Hand were nearly separated. Reünion of one of the injured members.* By THE EDITOR.

The patient was a bright little German boy, aged about 9 years, who, in playing with a hay-cutting machine, accidentally caught his hand in the knives, nearly cutting off the index and middle fingers. He was immediately carried home and I saw him about three quarters of an hour after the accident. The hæmorrhage had not been very profuse, and when I arrived, had entirely ceased. On examination I found a clean cut through the middle of the distal phalanx of both fingers, extending entirely through the bone and leaving them attached only by a strip of integument about a quarter of an inch wide. Taking into consideration the small amount of integument which is necessary to sustain the vitality of the end of the finger in such injuries, and the fact that even when entirely detached, the portion cut off will occasionally unite, it seemed advisable to make an attempt to save them. I accordingly brought the ends to their places by three points of the interrupted suture, and supported them by splints of narrow pasteboard, confined by a narrow roller, which was quite loosely applied, the ends of the fingers being left uncovered. The whole hand was then confined by means of another roller, and placed in a sling, the patient put to bed and the strictest quiet enjoined.

I saw the patient again on the same day, about eight hours after the dressing. The end of the index finger had a good color and was abundantly sensitive; but the middle finger was slightly discolored. The index finger continued the same, and the discoloration of the middle finger progressively increased until the third day, when the dressings were removed and new ones applied. The end of the index finger was then pretty firmly attached and of its natural color, but the middle finger was nearly black, though it also was pretty firmly attached to the stump. Sensation continued, howhowever, to some extent, in the middle finger up to Thursday, when it was amputated at the distal articulation. The stump healed kindly in a few days; the union of the index finger was complete.

This case seems interesting and instructive, as showing how slight a tegumentary connection is necessary in order to sustain the vitality in a part nearly removed from the body. It can soon be determined whether we will be able to save the finger in such a case. In the case of the index finger, which united, the normal circulation and sensation were established in a few hours, while the middle finger soon began to be discolored. In removing a finger at the distal articulation, it is always advisable to make the flap from the palmar surface and be sure to remove every portion of the matrix of the nail, in doing which it is necessary to make the dorsal incision some distance beyond the root.

